# Classification of GLOSSECTOMIES: Proposal for tongue cancer resections

**DOI:** 10.1002/hed.25466

**Published:** 2019-01-02

**Authors:** Mohssen Ansarin, Roberto Bruschini, Valeria Navach, Gioacchino Giugliano, Luca Calabrese, Fausto Chiesa, Jesus E. Medina, Luiz P. Kowalski, Jatin P. Shah

**Affiliations:** ^1^ Head and Neck Department European Institute of Oncology Milan Italy; ^2^ Plastic Reconstructive Surgery Department European Institute of Oncology Milan Italy; ^3^ Head and Neck Department Ospedale di Bolzano Bolzano Italy; ^4^ Department of Otorhinolaryngology The University of Oklahoma Health Sciences Center Oklahoma City Oklahoma; ^5^ Department Otorhinolaryngology‐Head and Neck Surgery Centro de Tratamento e Pesquisa Hospital do Cancer A.C. Camargo São Paulo Brazil; ^6^ Head and Neck Service MSKCC New York New York

**Keywords:** classification, glossectomy, TNM, tongue cancer, tongue surgery

## Abstract

**Background:**

Surgery of tongue tumors includes different procedures ranging from mucosal resection to complex combined resection. Numerous terms have been used to describe such procedures, but there is no consensus between the terminology and the extent of resection.

**Methods and Results:**

We searched the medical literature and found a lack of published information. We undertook to describe a new classification of surgical procedures for tongue tumor resection. We based it upon the surgical anatomy of the tongue and the spread of the cancer. We posited that there were five major types of glossectomy embracing all the methods of tongue cancer resection. This classification was reviewed and endorsed by an international team of experts.

**Conclusion:**

We propose a more precise classification than that currently in practice, thereby bringing clarity and consistency to the terminology, facilitating shared communication between surgeons, comparison between published research, and ultimately improving surgical practice and patient care.

## 1 INTRODUCTION

The term “glossectomy” is used to describe a variety of surgical procedures for the resection of tongue tumors. In 1978, Péri described four types of transoral glossectomy for noncancer‐related diseases of the tongue to reduce tongue volume.^1^ Later on, other attempts were made to classify macroglossia‐related surgery^2^ and tongue cancer‐related surgery.^3^

However, as yet, no classification exists for defining the extent of tongue resection for tumor removal. A literature search conducted on PubMed (National Library of Medicine, Bethesda, Maryland) using the terms “glossectomy” and “tongue cancer” yielded over 700 English‐language papers on surgery of the tongue for malignant neoplasms in the human. A thorough examination of the terminology used to define surgical removal of tongue cancers identified the following expressions: glossectomy, partial glossectomy, hemiglossectomy, subtotal glossectomy, transoral glossectomy, total glossectomy, cuneiform glossectomy, and compartmental glossectomy.

For the vast majority of these publications, there is no correlation between the term describing the surgical procedure and the extent of the resection carried out: for example, when the terms “subtotal glossectomy” or “hemiglossectomy” are used it is never specified what part of the tongue was removed and what was saved. The lack of a clear anatomical and functional definition of the various glossectomies reported in the literature leads to much confusion and misunderstanding. Such a degree of vagueness in the definition of tongue surgery makes it somewhat difficult to teach and train surgeons, and more difficult still to compare the experiences of different surgeons.

**Figure 1:**
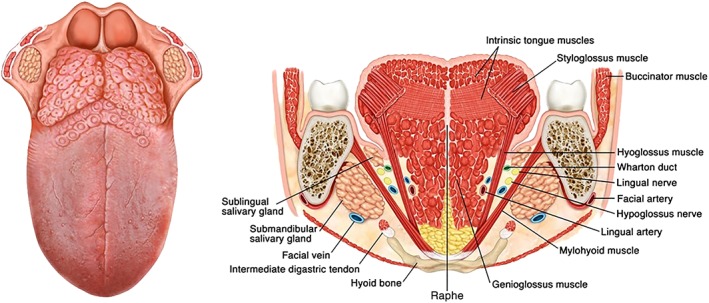
Tongue anatomy [Color figure can be viewed at wileyonlinelibrary.com]

The tongue can be considered as a median organ made up of two equal parts, separated from each other by the median raphe. Each half has its own extrinsic muscles (hyoglossus, styloglossus, genioglossus, and palatoglossus) and intrinsic muscles (longitudinal and transverse), venous and arterial vessels, and its distinct motor and sensory nerves (Figure 1).

Over time, the rationale and technical modalities of resection of tongue cancer underwent evolution. In the last century, most of the early and intermediate staged tongue cancers were treated by a partial glossectomy with margins at about 0.5‐1 cm from the macroscopic limits of the tumor.^4^ At the beginning of this century, the more frequent approach was a wide resection with larger free macroscopic margins (1.5‐2 cm). 5 Today we can better plan how to perform tongue tumor removal as a result of improved knowledge of the manner of superficial and deeper local tumoral spread along the muscles, the nerves, and the vessels,^6^, ^7^, ^8^, ^9^, ^10^, ^11^, ^12^ improved imaging techniques,^13^, ^14^ which allow surgeons to evaluate the involvement of each tongue muscle, and the new eighth edition American Joint Committee on Cancer (AJCC) TNM staging system.^15^, ^16^, ^17^ From a functional point of view, we should also underline that tongue resection often partially cut the muscles in such way that they lose their function.^18^, ^19^

It follows that surgical treatment of tongue cancers should be planned according to these clinical, pathological, and imaging findings. Early stages can usually be approached via the transoral route as the affected tissue grows and develops on the tongue mucosa or within the intrinsic muscles, and therefore, can be radically excised without necessitating extra‐oral access. When, however, the tumor affects one or more extrinsic muscles which have extralingual insertions, it is necessary to perform an en bloc combined transoral and lateral cervical approach to remove the muscles in toto from their insertions.^19^, ^20^, ^21^, ^22^, ^23^

Accurate preoperative evaluation, a precise analysis of the MRI or CT scan images, and staging using eighth edition of AJCC/Union Internationale Contre le Cancer (UICC)/TNM classification is therefore essential.

Starting from the cancer and from the muscles involved, we have developed a classification of glossectomies that clearly and immediately highlights what structures are involved and therefore which type of glossectomy is indicated for achieving a complete removal with minimal functional impact.

The aim of this article is to propose a terminology for tongue cancer surgery that is uniform and consistent.

We understand that any attempt at classification, especially in the field of tongue surgery, will inevitably be subject to a certain imprecision. A correct classification which not only takes into account all possible variables yet is also sufficiently simple to apply allows a standardized definition of each tongue resection. This is also very useful for education and training, sharing information, and comparing results.

This classification developed at the European Institute of Oncology, with long‐term experience in the surgical management of tongue cancer, was reviewed and endorsed by experts from the Memorial Sloan Kettering Cancer Center in New York, The University of Oklahoma Health Sciences Center, United States of America, as well as the A. C. Camargo Cancer Center in Sao Paulo, Brazil. It is based on very precise anatomical and functional components, whose removal depends on the precise nature of the tumor and the surgical anatomy of the tongue; however, to be practical and accepted, it should include the agreement of other surgeons.

## 2 TYPE I GLOSSECTOMY (MUCOSECTOMY)

Definition: Incision of the mucosa in healthy tissue with appropriate safety margins (1.0‐0.5 cm depending on whether or not the lesion is well defined). The mucosa and submucosa are included up to the intrinsic muscle fibers of the tongue. The deep resection margin should include a thin layer of the intrinsic muscles because of a possible invasion of the submucosa. Generally, the wound is left to heal by secondary intention, although the defect may be partially closed primarily or covered with a skin graft.

**Figure 2:**
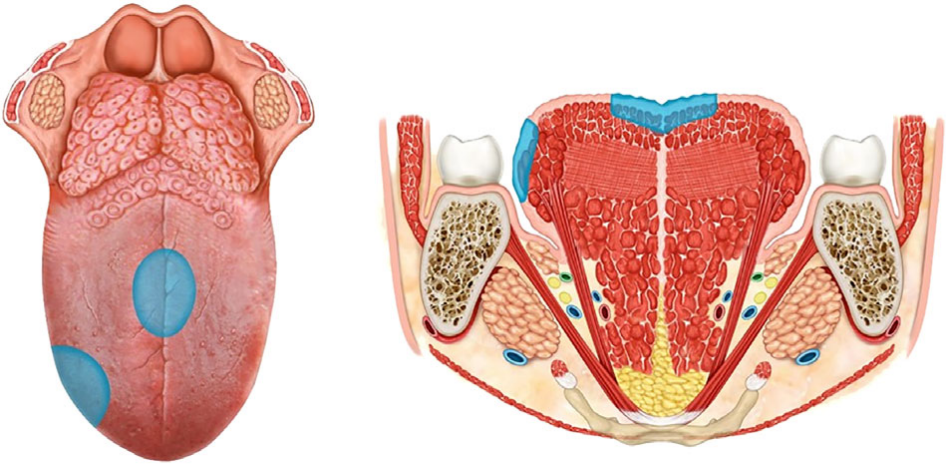
Type I glossectomy [Color figure can be viewed at wileyonlinelibrary.com]

Indication: Precancerous, superficial suspicious lesions, limited to the epithelium of the tongue without previous biopsy. The aim of surgery is to remove all the lesion with adequate margins up to the healthy tissue with both diagnostic and curative intent (Figure 2).

## 3 TYPE II GLOSSECTOMY (PARTIAI GLOSSECTOMY)

Definition: It includes the lesion and adjacent normal mucosa, submucosa, and the intrinsic muscles up to the surface of the extrinsic muscles (when the directions of the muscle fibers change), with appropriate safety margins (approximately 1.5 cm). The resection usually is diamond shaped on the surface, while more deeply, it is shaped like a truncated cone with the intrinsic muscles as the apex. The terminal branches of the lingual artery should be ligated and the lingual nerve is usually preserved. Closure may be partial or total with the objective of avoiding bleeding, postoperative edema, and retracted scars.

**Figure 3:**
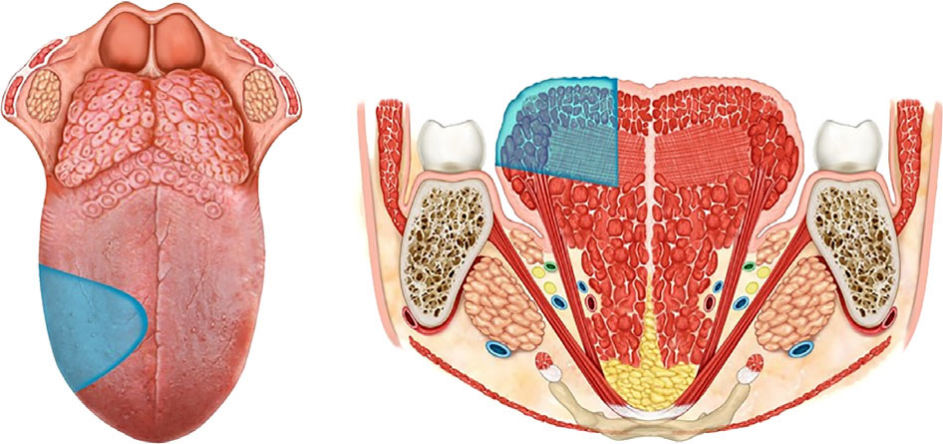
Type II glossectomy [Color figure can be viewed at wileyonlinelibrary.com]

Indication: Lesions infiltrating submucosa and superficially into intrinsic muscles, but not extrinsic muscles, or infiltration less than 10 mm deep^15^ (Figure 3).

## 4 TYPE III GLOSSECTOMY

### 4.1 Type IIIa glossectomy (hemiglossectomy)

Definition: The specimen includes the mucosa, submucosa, and intrinsic and extrinsic muscles ipsilateral to the lesion. The mucosa is resected up to healthy tissue with appropriate safety margins (at least 1.5 cm); the lingual artery must be ligated and removed en bloc with the lingual and hypoglossal nerves, in the specimen of the primary tumor and neck nodes. The base of the ipsilateral tongue is preserved. The tip of the tongue can be preserved or not.

**Figure 4:**
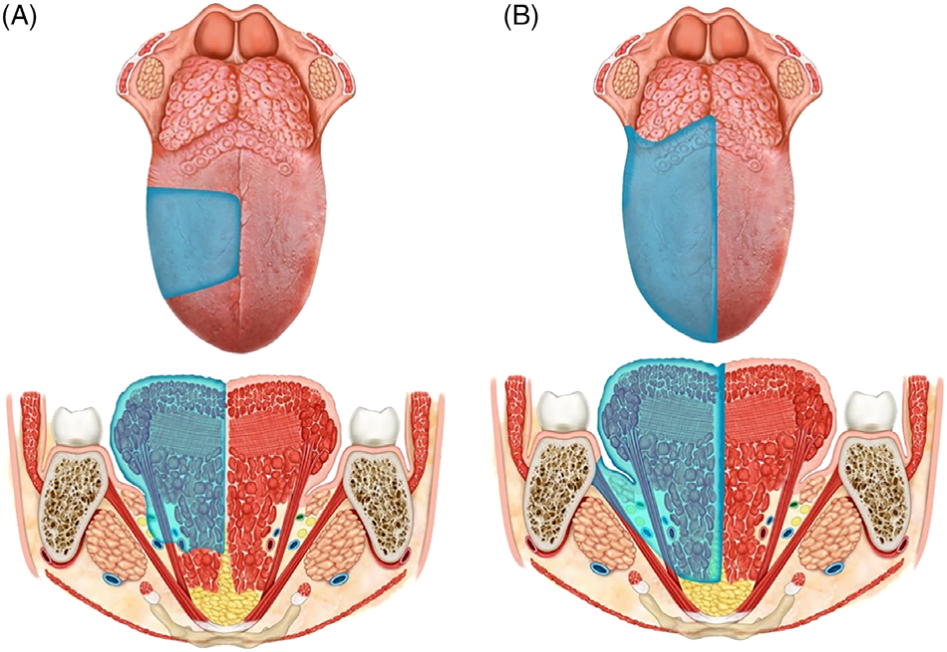
A, type IIIa glossectomy (hemiglossectomy). B, type IIIb glossectomy (compartmental hemiglossectomy) [Color figure can be viewed at wileyonlinelibrary.com]

Indication: lesions infiltrating the intrinsic and minimally extrinsic muscles or infiltration greater than 10 mm but confined within the ipsilateral tongue^15^ (Figure 4A).

### 4.2 Type IIIb glossectomy (compartmental hemiglossectomy)

Definition: The specimen includes the mucosa, submucosa, intrinsic and extrinsic muscles ipsilateral to the lesion, genioglossus, hyoglossus and styloglossus muscles, and the inferior portion of the palatoglossus muscle. Medially, the midline raphe is included in the resection. The lingual nerve is resected as far cranially as possible. The hypoglossal nerve is removed after the ansa, the lingual artery and vein is ligated in proximity to the horn of the hyoid bone, and removed en bloc with specimen and neck nodes.

Indication: Lesions massively infiltrating the intrinsic and extrinsic muscles but confined to the ipsilateral tongue (Figure 4B).

## 5 TYPE IV GLOSSECTOMY

### 5.1 Type IVa glossectomy (subtotal glossectomy)

Definition: This is an anterior subtotal glossectomy with preservation of both sides of the base of the tongue, posterior hyoglossus muscle, and hypoglossal and lingual nerves, from the less involved side.

**Figure 5:**
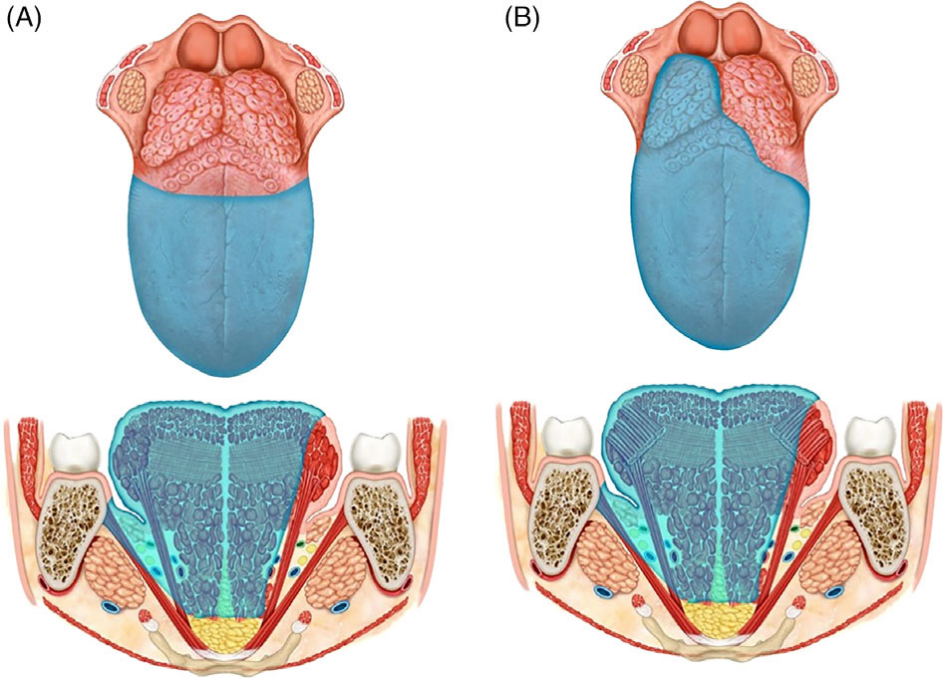
A, type IVa glossectomy (subtotal glossectomy). B, type IVb (near total glossectomy) [Color figure can be viewed at wileyonlinelibrary.com]

Indication: Lesions that arise in the anterior portion of the mobile tongue and exceed the hemilingual area of origin involving the contralateral genioglossus muscle but limited to mobile tongue (Figure 5A).

### 5.2 Type IVb (near‐total glossectomy)

Definition: Type IVa glossectomy with extension to the ipsilateral base of the tongue. The following contralateral structures are preserved: hyoglossus and styloglossus muscles, hypoglossal and lingual nerves, and lingual artery (functional unit of the base of the tongue).

Indication: Massive lesions that exceed the border of the hemilingual area of origin infiltrating the ipsilateral base of the tongue and the contralateral genioglossus muscle (Figure 5B).

## 6 TYPE V GLOSSECTOMY (TOTAL GLOSSECTOMY)

Definition: The specimen includes all of the mobile tongue and the base of the tongue transected at the level of the vallecula; it includes intrinsic and extrinsic muscles, both lingual arteries, hypoglossal, lingual nerves, and the floor of the mouth.

**Figure 6:**
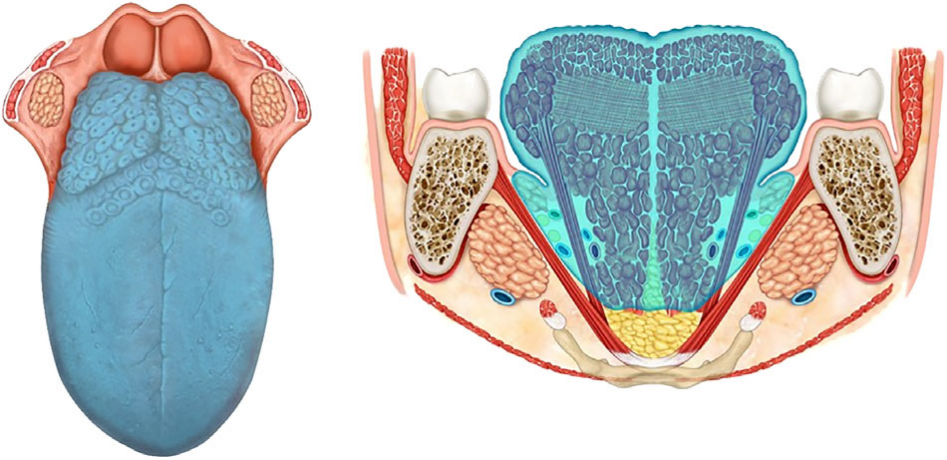
Type V glossectomy (total glossectomy) [Color figure can be viewed at wileyonlinelibrary.com]

Indication: Massive infiltrating lesions, for instance, those of the anterior ventral surface of the tongue, dorsum of the tongue, or the tongue base, which bilaterally involve the extrinsic genioglossus, hyoglossus, and styloglossus with impairment of the mobility of the tongue (Figure 6).

Depending upon the extent of the lesion, type III‐V glossectomies can be extended to some of the adjacent structures such as the geniohyoid muscle, digastric muscle, the epiglottis, all the larynx, the lateral wall of the pharynx, or the mandible. In such cases, the type of resection should be termed “glossectomy type extended to…” When, however, the type of glossectomy envisages that a structure be resected, but in fact the structure is preserved, the terminology applied in such a case should be “glossectomy type…” with the additional designation “with preservation of…”

## 7 DISCUSSION

The tongue is an organ of communication, speech, and articulation and is the principal structure that shapes and controls the food bolus during chewing and swallowing. It is a complex muscular structure covered with a specialized mucosal layer, anatomically comprising twin structures such as muscles, veins, arteries, and a nerve supply that join together in the lingual septum.

From the surface downward, we find the mucosa, submucosal, and muscular layers which include the intrinsic muscles, whose fibers have insertions only into the tongue itself, and the extrinsic muscles that arise from adjacent anatomical structures (genial tubercle of the mandible, hyoid bone, and styloid process).

Neoplasms can affect one or more of the above‐mentioned layers and the TNM classification of malignant tumors, the most commonly used cancer staging system, classifies them on this anatomical basis. The eighth edition of the AJCC/UICC TNM classification divides tongue tumors into early, intermediate, and advanced stages according to the depth of invasion of one or more of the four layers, from the surface inward, in addition to surface dimensions.

We realized the need for a shared and unified terminology, on re‐examining our own case records, in which we discovered that a range of different terminologies had been employed to refer to the very same surgical interventions. To clarify such confusion, we decided to base our proposal on the surgical anatomy of the tongue.

Other classifications of surgical procedures are reported in the literature, not only in the field of head and neck surgery but also in other fields in an attempt to create a common unifying language and to make such techniques more reproducible and comprehensible.^24^, ^25^, ^26^, ^27^, ^28^, ^29^, ^30^, ^31^, ^32^, ^33^Some of these classifications have entered into daily clinical practice, while some others less so. An early example is that of the Viennese surgeon Theodor Billroth, who in 1881 proposed the first classification of a surgical procedure for gastric resection.^26^ In the year 2000, the European Laryngological Society proposed a classification in which several terms relating to cordectomy were defined. This classification has now entered the clinical‐scientific language of laryngologists and is commonly used in the description of endoscopic laryngeal surgery.^27^ In 2012, Spiro et al^28^ proposed a classification of maxillectomy. Later Bidra et al^29^ and Brown et al,^30^ respectively, proposed the classifications of maxillectomy and mandibulectomy defects. More recently, the European Laryngological Society also proposed a classification for open horizontal laryngectomies.^31^ In 2016, the European Salivary Gland Society introduced a new classification for parotidectomies,^32^and more recently Turri‐Zanoni et al^33^ proposed a comprehensive classification system for transnasal endoscopic partial maxillectomy.

Considering the current wide acceptance of “compartmental” surgery for tongue cancer,^18^ it seemed timely to propose this classification for defining each procedure employed for the removal of tumors of the tongue.

The purpose of this work is not to enter into the merits of the indications of the neck dissection. Nor does it aim to explore the details of the reconstructive techniques used to repair the surgical defect. We limit ourselves to underline that, in general, type I glossectomy should not require prophylactic neck dissection. In type II glossectomy, supraomohyoid neck dissection should be carried out based on the probability of occult metastases and according to the institutional and international guidelines. From the type III glossectomy onward, the dissection of the neck should be done as an en bloc procedure. In the type IV and V glossectomies, the en bloc resection should be performed with bilateral neck dissection. In brief, the extent of tongue resection—type of glossectomy—is a reflection of the three‐dimensional extent of the primary tumor, and thus would entail the need for elective neck dissection based on the rising risk of occult metastases in the clinically negative neck.

The objective of this classification is to provide precise anatomical coordinates which will enable the techniques to be uniformly standardized, thereby rendering them repeatable and comparable. This will also provide the pathologist with detailed anatomical information. We believe that this kind of classification could help us even in the planning of the reconstructive time (the choice of the flap will be easier if, for example, one can readily predict a type IIIb, IV, or V glossectomies).

## 8 CONCLUSION

Based on our own experience and the published literature regarding the various types of glossectomy employed in tongue cancer surgery, we wish to propose a more precise and informative classification than what is currently in practice. It is based upon the surgical anatomy of the tongue which comprises also the routes of spread of the tongue cancer. Our proposal is to classify the surgical procedures for tongue cancer into five types, on the basis of well‐defined structures to be excised. The terminology describing each type of glossectomy must also indicate the surgical access employed.
